# Prompting a Fresh Start for Adults With Food Insecurity and Increased BMI: A Case Series of Four Patients in a Food Prescription Program

**DOI:** 10.7759/cureus.13857

**Published:** 2021-03-12

**Authors:** Jessica B Oliveira, Lennie To, Yazmine De La Cruz, Gregory W Schneider

**Affiliations:** 1 Humanities, Health, and Society/Family Medicine, Florida International University, Herbert Wertheim College of Medicine, Miami, USA

**Keywords:** community health, food insecurity, food prescription program, health education, nutrition, healthy foods, fresh produce, diet education, obesity, social determinant

## Abstract

Estimates place low intake of fruits and vegetables, physical inactivity, and high BMI (overweight-obesity) as all in the top 12 causes of death. Food and dietary education are becoming a focus in how we approach disease prevention and management, and food prescription programs in particular are showing promise, especially in under-resourced, food-insecure communities. This paper describes a pilot food prescription program in a handful of uninsured patients enrolled in an interprofessional clinical and educational program of a medical school in South Florida. This case series of four patients struggling with food insecurity profiles the demographic and clinical characteristics of the participants and provides the results of standardized assessments of their dietary behaviors, physical activity levels, and attitudes toward food before and after the intervention.

This four-month pilot food prescription program, Fresh Start Food Rx, involved a prospective case report of four patients seen on a mobile health center (MHC) for uninsured patients in South Miami, Florida. The MHC is part of an interprofessional health professions education, health care, and social service program of the Herbert Wertheim College of Medicine at Florida International University called the Neighborhood Health Education Learning Program (NeighborhoodHELP). A systematic review of South Miami MHC patient electronic medical records identified eligible participants for the program: patients with food insecurity and a BMI >30, with comorbid health conditions. Patients with greater BMI and more comorbidities were prioritized. Once enrolled, we provided biweekly packages of fresh fruits and vegetables along with monthly dietary education to the participants. Key measures included self-reported fruit and vegetable consumption, attitude toward healthy eating, and level of activity. Pre- and post-intervention focus groups assessed barriers the participants faced to eating healthy and pursuing physical activity, satisfaction with the program, feedback on strengths and weaknesses, and anticipated behavioral changes after completion of the program.

Prior to the intervention, participants reported eating fruits on an average of 4.5 days out of the week. Post-survey answers increased to 5.0 days per week. Though the average amount of days per week that participants reported eating vegetables decreased slightly, the average number of vegetable servings that participants reported eating in a week increased. At termination of the program, most participants agreed that a diet rich in fruits and vegetables is good for you, that it is important to eat fruits and vegetables every day, and that a diet rich in fruits and vegetables can protect against cancer.

This case study demonstrates that easier access to healthy foods, such as fresh produce delivery, and regular health education have the potential to promote healthier attitudes toward foods like fruits and vegetables. This change in attitude can then influence behavior, such as choosing to try new produce or increasing the amount and frequency of produce consumption. With the lessons learned from this small pilot program, the authors helped facilitate the expansion of a larger food prescription program in conjunction with a community partner hospital in the area. Findings from this experience might prove useful for others attempting to develop or expand a food prescription and health education program of their own.

## Introduction

When looked at from the perspective of preventable causes of death in the United States, smoking and hypertension are responsible for the largest number of deaths, but other dietary, lifestyle, and metabolic risk factors for chronic disease cause a substantial number of deaths. Estimates place high BMI (overweight-obesity), low intake of fruits and vegetables, and physical inactivity all in the top 12 causes of death [1]. Elevated BMI, in particular, continues to be a public health crisis in the United States, accounting for 18% of deaths among Americans ages 40-85 [2]. In general, the highest rates of obesity are found in Latino, African-American, and lower-income populations, and in those without a college degree [3]. Poor families in many communities are more affected due to lack of resources for healthy eating and the increased presence of convenience stores or fast food restaurants that sell affordable, calorie-dense food, which is of lower nutritional value [4]. Food insecurity exists whenever the availability of nutritionally adequate and safe foods or the ability to acquire acceptable foods in socially acceptable ways is limited or uncertain [5]. Clusters of obesity are found in many food-insecure areas that have been termed “obesogenic environments” because of the shared role of environment and behavior on obesity. Obesity is born from “the sum of influences that the surroundings, opportunities, or conditions of life have on promoting obesity in individuals or populations” [6].

Food and dietary education are becoming a focus in how we approach disease prevention and management. Progress with traditional drug therapies has been slow regarding food-related chronic illnesses. Management of diet-dependent chronic diseases like obesity, diabetes, and hypertension becomes even more challenging in low-income, food-insecure communities. Newer models consider the importance of mindful eating practices and awareness of behavioral patterns. The Fresh Prescription Program in Detroit, run by Omar, Heidemann, and colleagues, enrolled participants from an outpatient internal medicine clinic. Participants completed nutrition educational counseling sessions and were able to redeem their “prescriptions” at local farmer's markets or with boxed food deliveries in fresh produce. Seventy-one percent of participants reported being better able to manage their health conditions after participating in the program and 44% were able to decrease the number of times they ate unhealthy food during an average week [7]. 

There is a complex and important relationship between socioeconomically vulnerable populations, food insecurity, and chronic diseases. The public health consequences of sedentary lifestyles result from the interplay of dietary behaviors, physical activity, lack of access to healthy foods, health literacy, and other mental and physical health conditions. Food choices are influenced by many factors, perhaps most importantly availability [8], and availability is part of a larger systemic process. A prescription for “healthy food” may be a promising approach for both influencing individual behaviors and for engaging community stakeholders in broader solutions. Such interventions can serve as a platform for community-based interventions to address food behaviors and physical activity. This paper describes a four-month pilot food prescription program in a handful of uninsured patients enrolled in an interprofessional clinical and educational program of a medical school in South Florida. This case series of four patients struggling with food insecurity profiles the demographic and clinical characteristics of the participants and provides the results of standardized assessments of their dietary behaviors, physical activity levels, and attitudes toward food before and after the intervention. As an exploratory study, it offers valuable insights for those considering launching or expanding a food prescription program. 

## Case presentation

Participants 

Participants included four patients who were seen on a mobile health center (MHC) for uninsured patients in South Miami, Florida. The MHC is one part of a multidimensional health professions education, health provision, and social service program associated with the Herbert Wertheim College of Medicine at Florida International University called the Neighborhood Health Education Learning Program (NeighborhoodHELP). The primary care provider on the MHC identified potential participants based on patient characteristics.  MHC patients who met the clinical inclusion criteria, based on BMI and on food insecurity, were eligible.  The inclusion criteria included patients who had food insecurity plus a BMI of more than 40, or a BMI of more than 35, with two or more comorbid conditions: hypertension, diabetes, hyperlipidemia, steatohepatitis, coronary artery disease, or obstructive sleep apnea.  Food insecurity was identified during routine bimonthly screening by the Outreach NeighborhoodHELP team. Systematic review of the electronic medical record identified patients seen on the MHC who met the BMI and other clinical criteria.  MHC primary care providers reviewed the generated list and prioritized patients with greater BMI and heavier burden of comorbidities.  These patients were recruited one-by-one down the prioritized list, until 10 patients were accepted.  Recruitment consisted of a standardized brochure describing the program and a phone call from one of the clinical personnel on the MHC who has Health Insurance Portability and Accountability Act of 1996 clearance and who routinely contacts patients offering them services as part of NeighborhoodHELP. Of the 10 patients who gave verbal consent during the recruitment phase, only four patients presented for the initial food provision and education session. This article summarizes the experience of these four participants. 

Participant 1 was a 32-year-old uninsured woman, suffering from depression. Her BMI was 32.29. She lived in a three-bedroom condominium with six other family members and worked at night so that she could care for her child during the daytime. She afforded rent through a subsidized housing program and through the contributions of family members. She described herself as Haitian American. 

Participant 2 was a 56-year-old woman with hypertension, hyperlipidemia, and chronic knee pain. Her BMI was 43.61. During the course of the intervention, she lost her job of 38 years at a department store that went bankrupt and lived in subsidized housing with her husband and a grandson for whom she provided care. She identified herself as African American. 

Participant 3 was a 30-year-old uninsured woman with pre-diabetes. Her BMI was 38.84. She lost her employment shortly after enrolling in the program and was supported by six other family members who lived with her in a small three-bedroom apartment. She described herself as Haitian American. 

Participant 4 was a 65-year-old woman with pre-diabetes, osteopenia, gastritis, hyperlipidemia, and hypertension. Her BMI was 32.21. She was unemployed and lived with her daughter’s family in a three-bedroom townhome, where she helped care for her grandchildren. She considered herself Hispanic. 

Measures and Intervention**

As part of providing primary care services and chronic disease management, NeighborhoodHELP also attempts to mitigate the social determinants of health, including food insecurity. The target population for the Fresh Start Food Rx Program included patients with both food insecurity and elevated BMI, that is, those who might benefit from the healthy food provided and from the monthly health education sessions. The program provided biweekly packages of fresh fruits and vegetables and monthly dietary education to four participants from the MHC.   

The data collection consisted of two main components: focus groups and pre- and post-intervention surveys.   

The *focus groups* with all four participants occurred at both the outset and conclusion of the four-month program. The program began with the informed consent process and then proceeded into the initial focus group. The ½-hour focus group was part of the 2-hour group session that launched the program. This pre-intervention focus group involved questions related to where the participants obtained their food and if there were any barriers they faced to eating healthy and pursuing weekly physical activity. The post-intervention focus group occurred as part of the final 2-hour group session that ended the program. This post-intervention focus group, also ½ hour in duration, assessed participants’ satisfaction with the program, asked for feedback on the strengths and weaknesses of the program, and queried participants about any anticipated changes in their behavior after completing the program.   

The *pre- and post-intervention surveys* were completed as part of the initial and final group sessions as well. These surveys consisted of standardized questions related to food insecurity, behaviors related to eating fruits and vegetables, and weekly physical activity levels.   

The health intervention consisted of biweekly distribution of fresh fruit and vegetable boxes to the enrolled participants. The participants either received the food boxes during the monthly health education session at a local community center or via a delivery to their homes, which occurred at the alternating two-week intervals.    

Study Objectives**

Objective 1: To encourage behavioral change by increasing consumption of fruits and vegetables among participants. We assessed for evidence changes in intake via self-report on pre- versus post-intervention surveys. This was done by asking participants to track the number of days they ate fruits and vegetables in a week, as well as the number of servings of fruits and vegetables consumed per day. We looked for patterns for each participant before and after the intervention and looked for changes among the participant’s behavior and dietary habits. 

Objective 2: To foster more positive attitudes toward fruits and vegetables among participants. We aimed to promote positive changes in attitude toward healthy foods through the monthly health education sessions. We assessed for evidence of these changes in attitudes via Likert scale scores on pre- versus post-intervention surveys. These included questions that assessed participants’ attitudes such as enjoyment of new foods, and that a diet of fruits and vegetables is good for you. Participants’ attitudes toward fruits and vegetables were compared before and after intervention for themselves, as well as overall changes between the group of participants. Please see Table 1. 

**Table 1:** Attitudes Toward Food SD – Strongly disagree; D – Disagree; N – Neutral; A – Agree; SA – Strongly agree; NA – Answer not available

Response	Participant 1		Participant 2		Participant 3		Participant 4	
	Pre	Post	Pre	Post	Pre	Post	Pre	Post
“I enjoy trying new foods”	SA	SA	N	N	N	D	N	SA
“I would taste a new fruit”	SA	A	N	N	A	D	A	SA
“I would taste a new vegetable”	SA	N	N	N	N	N	A	SA
“Diet of fruits/veg is good for you”	N	SA	SA	SA	SA	NA	SA	SA
“Importance to follow recommendation”	N	N	N	N	N	N	N	N
“Rich diet protects against cancer”	SA	A	N	SA	SA	D	SA	SA

Objective 3: To reduce barriers to healthier eating among participants by providing access to healthy foods and linking participants to community resources. As the participants in this program experienced food insecurity, we attempted to reduce barriers to healthy eating by providing the participants with a biweekly distribution of fruits and vegetables. Our focus groups assessed the participants’ perceptions of perceived supports and barriers to eating healthier food and to pursuing regular physical activity. 

Self-Reported Vegetable and Fruit Consumption   

**Figure 1:**
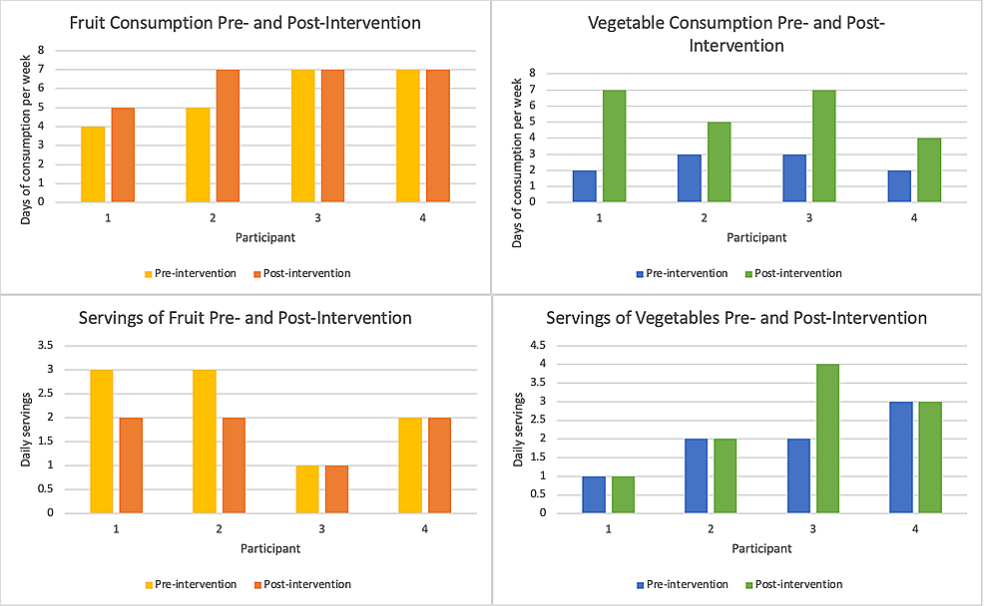
Self-Reported Fruit and Vegetable Consumption Pre- and Post-Intervention

**Figure 2:**
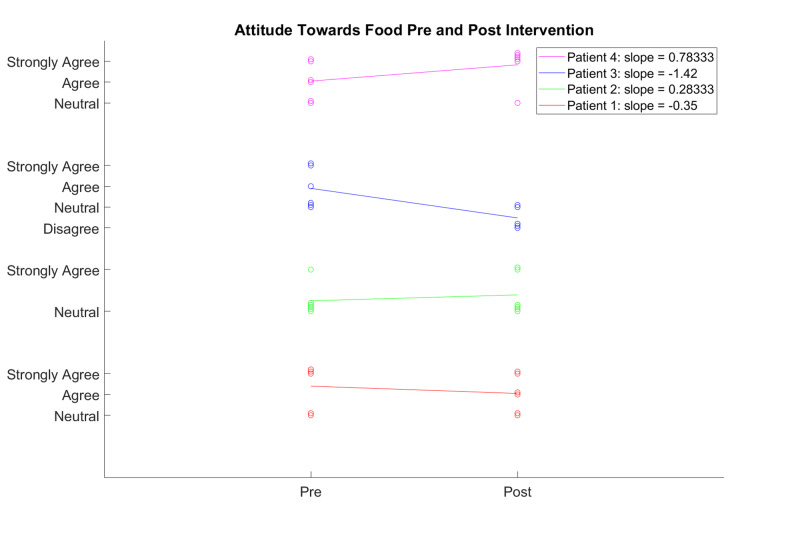
Trends in Attitude Toward Fruits and Vegetables Pre- and Post-Intervention

All of the frequencies cited come from participant self-report.  At the beginning of the program, participants reported eating fruits on an average of 4.5 days out of the week. Post-survey answers demonstrate a slight increase in the fruit consumption per week, including an increase of servings per day.  Self-reports regarding vegetable intake showed a different pattern.  The participants reported eating vegetables fewer days per week; however, they did report eating larger servings of vegetables per day.  Please see Table 2, Figure 1, Table 3, and Figure 2*.*

**Table 2:** Self-Reported Fruit and Vegetable Consumption

	# of days of eating fruit per week		# of servings of fruit per day		# of days of eating vegetables per week		# of servings of vegetables per day	
Participant number	Pre	Post	Pre	Post	Pre	Post	Pre	Post
1	4	5	3	3	7	7	1	2
2	5	7	2	2	7	7	1	2
3	2	3	1	2	3	2	2	3
4	7	5	1	2	7	4	4	3
Mean	4.5	5	1.75	2.25	6	5	2	2.5

**Table 3:** Change in Fruit and Vegetable Consumption

Response	Participant 1	Participant 2	Participant 3	Participant 4
# of days of eating fruit per week	Increase	No change	No change	Increase
# of servings of fruit per day	Increase	No change	No change	Increase
# of days of eating vegetables per week	Increase	Increase	Decrease	Decrease
# of servings of vegetables per day	Decrease	Increase	Decrease	Decrease

Attitudes Toward Food**

Half of the participants reported no change in attitude toward enjoyment with trying new foods. One participant showed a positive change in enjoyment with trying new foods, whereas the other demonstrated a negative change in doing so. Only one participant reported a positive change in tasting a new fruit or vegetable, even if not knowing what it was. The rest of the participants answered with either no change or negative change in trying new fruits or vegetables that they are unfamiliar with. At the termination of the program, most participants agreed that a diet rich in fruits and vegetables is good for them. In both the pre- and post-intervention surveys, all the participants agreed that it is very important to follow the recommendation of eating fruits and vegetables every day. By the end of the program, most participants agreed or strongly agreed that a diet rich in fruits and vegetables can protect against cancer.  Please see Figure 2. 

## Discussion

The Fresh Start Food Rx Program was a four-month prospective case series, which explored the feasibility and effectiveness of a food prescription program for overweight and obese patients who struggled with food insecurity. The participants were recruited from HWCOM’s NeighborhoodHELP MHC in the city of South Miami, Florida. The purpose of the program was to promote healthier eating habits through monthly educational sessions and administration of boxes of fresh produce. In 2016, the Florida Department of Health reported that 64% of Miami-Dade County residents were either overweight or obese. The age-adjusted rate for heart disease in Miami-Dade was 148.5 per 100,000 [9]. Food insecurity plays a prominent role in the health outcomes of minority populations. According to a study conducted by Berkowitz and colleagues, low-income families have higher rates of chronic disease because of lack of availability of healthy food and access to exercise facilities [4].  One study conducted in Pittsburg determined a close relationship between neighborhoods with supermarkets and slower growth in metabolic diseases among the community population [10].  Food prescription programs can bridge the gap between struggling families and healthy food. For example, a food prescription program in Detroit proved to be effective with its participants; 44% of them reported eating less unhealthy food after receiving deliveries of boxed fresh produce [7]. The Fresh Start Food Rx program mimicked this model by providing boxes of fruits and vegetables biweekly for four months to each participant.   

Key measures collected were fruit and vegetable consumption, attitudes about healthy eating, and level of activity. Participants 1 and 2 reported more days of eating fruits and vegetables per week at the end of the program, whereas Participant 3 reported no change and Participant 4 reported a decrease. Similar patterns are apparent with reports of number of servings of fruits and vegetables eaten per day, with an increase for Participant 2 and a decrease for Participants 1, 3, and 4. One explanation for the decrease in servings could be difficulty in understanding what constitutes a serving size for a fruit versus a vegetable. Responses about attitudes among the participants demonstrate a positive or neutral change when asked about willingness to try to taste a new piece of produce and that a diet of fruits and vegetables is good for them. However, it is difficult to predict if these responses reflect long-lasting changes in attitude. 

The implementation and results of the Fresh Start Food Rx Program did not come without limitations. Due to a smaller sample size, generalizability of our survey results is not feasible. Our program demonstrated the difficulty in implementing community interventions. It was difficult even to obtain full buy-in from the initial participants. We had 10 patients who had given verbal agreement to participate in the program, but only four ended up participating. One other pilot program cited ongoing communication and reminders as a remedy to maintain client retention [11]. Our program gave reminders twice during the week of the upcoming educational session.  The first reminder was a midweek phone call, which mostly led to voicemail messages. The second reminder was in the form of a text and sent 24-48 hours before the visit. The four remaining participants suggested that the educational session time should be on weeknights instead of Saturdays because of time availability.  

As a further example, two participants explained that they did not know how to cook with some of the vegetables because they were unfamiliar. Participants suggested an exchange of recipes along with a description of each fruit and vegetable provided. Other food prescription programs cited proximity to the food pick-up site as a challenge to client retention [12,13]. However, our program addressed this problem through direct delivery of produce. Nonetheless, scaling a delivery system for a larger program could prove difficult without the appropriate level of funding and support staff. Additionally, future efforts with the program could include a stronger physical activity component with instruction on exercise routines integrated in each educational session.  

Our experience highlights the importance of taking into account social, economic, and cultural factors when designing a food prescription program. Diversity in experiences, cultures, attitudes, and zip codes can all affect individual preferences in food choices. Unexpected life events, such as a medical expense or costs of a car repair, can also lead to difficult choices, including selecting more economical but lower-quality food, contributing to the cyclic nature of food insecurity. Food may be more abundant at the beginning of the month when one is paid or food stamps are received, and then scarcer toward the end of the month when funds are low. Many food desert areas, particularly in disadvantaged urban populations, may also be "food swamps": “areas with a heavy concentration of fast food restaurants and convenience stores offering nutrient poor food” [14]. The PHRESH study in Pittsburg estimated the impacts on residents' economic status and health in two African American low-income, food desert neighborhoods. The researchers surveyed a randomly selected cohort in the two low-income neighborhoods before and about one year after the opening of a local supermarket. Supplemental Nutrition Assistance Program (SNAP) participation and food insecurity decreased in the neighborhood with the supermarket but not in the comparison neighborhood, where SNAP participation increased, and food insecurity was stable. In addition, the neighborhood with a supermarket saw evidence of slower growth in diagnosing high-cholesterol, arthritis, and diabetes incidence, relative to the comparison neighborhood [10]. 

The investigators believe that the success that we did have to some extent resulted from the fact that the program was part of NeighborhoodHELP, a much larger intervention designed to provide culturally competent care and to address the social determinants of health. This program strives to provide patient-centered and household-centered care that takes into account individual and household needs, priorities, and preferences. The Fresh Start initiative reveals how important it is to consider a wide variety of factors to best serve the needs of clients struggling with food insecurity. 

## Conclusions

Environment and behavior both play roles in shaping our health status and well-being. Some environments can contribute both to food insecurity and to high rates of obesity. Lack of access to healthy foods combined with lack of dietary education can make it difficult to thrive in these environments. This case series demonstrates that easier access to healthy foods, such as fresh produce delivery, and regular health education have the potential to promote healthier attitudes toward foods like fruits and vegetables. This change in attitude can then influence behavior, such as choosing to try new produce or increasing the amount and frequency of produce consumption. With the lessons learned from this small case series, the authors helped facilitate the expansion of a larger food prescription program in conjunction with a community partner hospital in the area. Findings from this experience might prove useful for others attempting to develop or expand a food prescription and health education program of their own.
